# Knowledge, Attitudes, Psychosocial Perspectives and Applied Epidemiology in the Control of Head Lice (*pediculosis capitis*) in Croatian Preschool Children: A Qualitative Study on Childcare Professionals and Health Coordinators

**DOI:** 10.3390/children9010066

**Published:** 2022-01-04

**Authors:** Marijana Neuberg, Ines Banfić, Tina Cikač, Rosana Ribić, Sanja Zember, Tomislav Meštrović

**Affiliations:** 1 University Centre Varaždin, University North, 42 000 Varaždin, Croatia; mneuberg@unin.hr (M.N.); inesbanfic@gmail.com (I.B.); ticikac@unin.hr (T.C.); rribic@unin.hr (R.R.); 2 Department for Urogenital Infections, University Hospital for Infectious Diseases Dr. Fran Mihaljević, 10 000 Zagreb, Croatia; zember.sanja@gmail.com; 3 Clinical Microbiology and Parasitology Unit, Dr. Zora Profozić Polyclinic, 10 000 Zagreb, Croatia

**Keywords:** parasitic disease, head lice, *pediculosis capitis*, *Pediculus humanus* var. *capitis*, outbreak, kindergarten, childcare professionals, health coordinators, preschool children, qualitative study

## Abstract

This study aimed to concurrently determine the perceived knowledge, attitudes and perspectives of childcare professionals working in kindergartens towards *pediculosis capitis*, a common ectoparasitic disease also known as head lice, as well as gain insights into procedures and control measures that are implemented in an outbreak setting. We used a qualitative approach with a problem-centered, semi-structured and three-part interview technique conducted in selected kindergartens of Varaždin and Međimurje counties of the Republic of Croatia. Based on a purposive (deliberate) sampling method, the study included both childcare professionals and on-site health coordinators aged between 21 and 56 years of age. Five main themes were put forth as a result of the conducted thematic analysis: prevention and control measures for managing head lice, information and knowledge, social issues, psychological issues and disease perception. Each of these themes also had specific emerging categories based on participants’ responses. Even though all respondents confirmed that the disease is continuously monitored only a few of them mentioned that a scalp examination was an inherent part of disease surveillance within the kindergarten community. Moreover, we found that information on *pediculosis capitis* is available to both parents and childcare professionals, but with a questionable uptake. Additionally, the majority of the respondents reported that parents tend to hide the infestation in their children due to shame and/or to avoid social stigma, and thus often fail to inform kindergarten teachers and health coordinators about the problem. In conclusion, our findings have implications for further practice and the introduction of tailored public health measures for the most vulnerable populations, most notably kindergarten children.

## 1. Introduction

The hematophagous parasite *Pediculus humanus* var. *capitis* (Anoplura: Pediculidae), commonly known as head louse (plural: head lice), represents a causative agent of *pediculosis capitis* and is among the most prevalent ectoparasites around the world. Head lice are famous for their distinctive adaptability to the human organism, as the body’s temperature and relative humidity create perfect conditions for their life cycle [1,2,3]. Akin to other blood-sucking insects, *Pediculus humanus* var. *capitis* bites the skin of the scalp in order to feed on human blood, and in turn produces various symptoms/signs (such as pruritus and eczematous changes) due to the irritative effects of its saliva [3,4].

Although the disease epidemiology can vary in accordance with societal and cultural behavior, *pediculosis capitis* due to head lice is classified as one of the six epidermal parasitic skin diseases, which can be viewed as an informal subcategory of neglected tropical diseases [5]. However, the majority of studies on head lice actually focus on disease prevalence and treatment effectiveness [1,6]; hence, the literature on the actions, experience and opinions of parents, health providers and preschool as well as schoolteachers is extremely scarce [6,7]. This is irrespective of the pervasive understanding that problems and difficulties in regard to *Pediculus humanus* var. *capitis* are predominantly of a social and psychological rather than a medical nature [1,2,3,6,7,8].

In addition, country-specific data are pivotal for planning any communicable disease control program, which is also valid for *pediculosis capitis* [1,9]. More specifically, local data can arguably improve control strategies for this disease; the value of local research endeavors to inform practical approaches was reinforced by a survey in South Africa that was carried out by the provincial communicable disease program, which showed that only children of European and Indian ancestry in a mixed-race school had pediculosis [10]—this is just one salient example. Even guidelines from various countries have highlighted the pivotal role of universities (but also other research institutions) in conducting research on various aspects of pediculosis in their own countries [8]. This is why there is a dire need for more local data to improve our understanding of the global context.

Ergo, the principal aim of this research was to examine the attitudes of childcare professionals and health coordinators working in kindergartens towards *pediculosis capitis*, i.e., head lice, by using a qualitative study design. To our knowledge this is the first study in the medical literature where the emphasis was placed not only on appraising the personal views of such specific professionals in regard to this infectious disease, but also on specific social and psychological issues, as well as their knowledge and the readiness for the introduction of specific preventative and control measures.

## 2. Materials and Methods

This research was conducted by the means of a qualitative approach using a problem-centered and semi-structured interview method in selected kindergartens of Varaždin and Međimurje counties in the Republic of Croatia during 2020. We have opted for such an approach since it can provide deeper, more nuanced and more effective insights into our principal research questions when compared to the survey method (which was thus far actually the most common method for researching issues concerning *pediculosis capitis*). Moreover, a qualitative methodology can also provide a steadfast and in-depth exploration of the ways of thinking and different viewpoints in our target population—childcare professionals and employed health coordinators in kindergartens.

### 2.1. Respondents

The selection of respondents for the study was based on a purposive or deliberate sampling method. Given the qualitative nature of the research, the length of the research process and current recommendations in determining sample sizes for studies akin to ours, the total number of childcare professionals in our study was set at fourteen. Institutional health coordinators also participated in the research, and their number was limited to three since some kindergartens from which the sample of childcare professionals was selected did not have a person employed as a health coordinator. Hence, the total number of our study participants was seventeen. The main inclusion criteria for our respondents were employment as childcare professionals or health coordinators, regardless of their educational attainment (i.e., the highest level of education that they have completed), and the length of service.

### 2.2. Data Collection Method—A Semi-Structured Interview

In line with our postulated research goal, a qualitative approach to collecting the necessary data was used. The semi-structured interview method provided flexibility in data collection, i.e., there was a predetermined structure that was the same for all respondents, but there was a possibility of creating and asking new questions and sub-questions as a result of the content presented by the respondent. Such a procedure of making sub-questions was used when respondents provided incomplete answers or had difficulty expressing themselves clearly.

The interview consisted of three groups of questions. The first group of questions referred to the socio-demographic characteristics of the respondents. In this group the respondents were asked a total of seven questions. The following data were collected for this purpose: sex, age, educational attainment, overall length of service and the group of children by age that the respondent was responsible for.

The second part of the interview consisted of sixteen problem-oriented questions that were related to the issue of *pediculosis capitis*. These were open-ended questions and the vocabulary was adapted to the respondents. The questions were focused on obtaining data on the procedures that would be undertaken in the event of the spread of head lice in kindergartens, gathering information from and giving information to parents, monitoring the disease in children, educational materials and educating both childcare professionals and parents. Furthermore, the questions were related to obtaining data on the frequency of *pediculosis capitis* in kindergartens within the last six months, the number of infected children in the group in which the respondent works and the procedures that would be undertaken when parents bring an infected child back to kindergarten.

The third part of the interview consisted of seven questions aimed exclusively at researching the attitudes of childcare professionals towards head lice infestation. Questions were asked about a connection between the occurrence of *pediculosis capitis*, poor hygiene habits and low socioeconomic status; in this way we wanted to obtain data on the attitudes of respondents towards this issue, given that even in developed countries head lice phenomena can be linked with the aforementioned variables. Furthermore, questions were asked about the actions and reactions of the respondents to children infected with head lice as well as personal actions and reactions when the respondents find out that there is a head lice infestation among the children in kindergartens. The last question offered the possibility of giving an extensive answer and expressing one’s view on the subject, which has been related to the previous group of questions.

### 2.3. The Interview Process

At the beginning of each interview the goal and purpose of the research were explained to each respondent. For simplicity and easier follow-up of the interview, the conversation was recorded with a voice recorder; the respondents had been previously informed about this and their specific consent was asked for. Furthermore, all respondents signed an informed consent form, thereby giving their consent to participate in the survey and stating that they were doing so without coercion. Each respondent was given the opportunity to withdraw from the research at any time. They were guaranteed anonymity and confidentiality; they were informed that the data would be used exclusively for research purposes.

After presenting the research goal and obtaining informed consent from the respondents the interviewer posed a set of predetermined questions according to a semi-structured interview for childcare professionals in kindergartens and health coordinators (as explained above). The time for the interview was limited to twenty to thirty minutes for childcare professionals in kindergartens and fifteen to twenty minutes for health coordinators. Depending on the situation the interviews were conducted with childcare professionals in a separate room in kindergartens (n = 10) or in an informal environment, e.g., at a person’s home or in a coffee shop (n = 4), and with health coordinators in a separate room in kindergartens (n = 2) or in an informal environment (n = 1).

### 2.4. Data Analysis

After conducting a semi-structured interview the recorded conversation was literally transcribed into a textual format and the obtained information was analyzed. The transcribed data were investigated inductively to generate categories and explanations in a process of thematic content analysis. Sentences and expressions belonging to a given respondent were enumerated by the authors. The data were processed to reduce the content to the level required and sufficient for research. The content was summarized, i.e., repetitions and digressions were deleted to obtain shorter, meaningful sentences; in the second round it was paraphrased into several key words that generalized and condensed the meaning and finally yielded qualitative data.

More specifically, the coding of the sentences was conducted by considering the sub-sections and theoretical grounds of our study approach. Two separate coding lists were utilized to evaluate the conceptual meaning found in the expressions and/or sentences; we then compared the lists for similarities and differences. By piecing together the codes (or formulated meanings) we formed the “themes” of our research, which enabled us to reach a consensus. Crucial expressions with their formulated meanings were put in adequate theme categories, while closely related themes were grouped together.

## 3. Results

### 3.1. Respondents and Main Themes

The age range of the respondents was from 21 to 56 years. In terms of the total number of childcare professionals there were thirteen female and one male respondents who participated in the research, one of whom held a master’s degree, twelve completed a bachelor’s degree in early childhood education and one had a bachelor’s degree in nursing. The overall length of service of the respondents ranged from 6 months to 25.5 years. The research participants were childcare professionals working in kindergartens in Varaždin and Međimurje counties, i.e., three respondents were from a kindergarten in Međimurje County and eleven respondents were from a kindergarten in Varaždin County. A demographic profile of the respondents is given in Table 1 (in order to preserve the anonymity of the respondents and facilitate data analysis capital letters, A–N, were assigned to each respondent).

In addition to childcare professionals, employed institutional health coordinators also participated in this study. The age range of the respondents was from 25 to 35 years. Three female respondents participated in the research, all of whom held a bachelor’s degree in nursing. The overall length of service of the respondents ranged from one to five years. Research participants were health coordinators working in kindergartens in Varaždin and Međimurje counties, i.e., one respondent from a kindergarten in Međimurje County and two respondents from a kindergarten in Varaždin County. A demographic profile of these respondents is given in Table 2 (in order to preserve anonymity of the respondents and facilitate data analysis capital letters with a number, A1–A3, were assigned to each respondent).

Five main themes were put forth as a result of the thematic analysis:

- Prevention and control measures for managing head lice;
- Information and knowledge;
- Social issues;
- Psychological issues;
- Perception of *pediculosis capitis*.

Categories within the main themes were created based on the recurrent content or responses that were completely different. The content of all categories in the tables is presented, starting with the most common and equal answers and ending with the content the respondents differed on.

### 3.2. Prevention and Control Measures for Managing Head Lice

The analysis of the theme “*Prevention and control measures for managing head lice*” revealed that most of the answers could be classified into four categories, namely: monitoring the disease within the kindergarten community, notice to parents of all children in kindergarten, scalp examination and isolation (Table 3).

For the category “monitoring the disease within the kindergarten community” we obtained data on the procedures used for monitoring children in all groups, regardless of the group in which this infectious disease first emerged. All respondents confirmed that the disease is monitored among all children in all groups, but only a few of the respondents mentioned that a scalp examination was included in the measures for monitoring the disease within the kindergarten community. More precisely, only three respondents said that a scalp examination was performed: one of them stated that it was done by a health coordinator and the other two stated that a scalp examination was performed by a health coordinator assisted by a childcare professional. This might be explained by the fact that five out of fourteen respondents stated that they did not have a person employed in their kindergarten as a health coordinator; four out of the remaining number of respondents stated that the health coordinator was not present all day in their kindergarten because some kindergartens have subsidiaries, meaning that several kindergartens share a health coordinator.

A very interesting answer was given by respondent A, whose answer to the question of whether there was a person currently employed as a health coordinator in the kindergarten where he/she currently works read: “*Officially, there is no such employee,* i.e., *our kindergarten employs two bachelors of nursing and one person with a high school diploma, who, if necessary, perform the duties of a health coordinator.*” This indicates a major problem in hiring health coordinators, and therefore a problem in addressing infectious diseases among children in kindergartens. Furthermore, such procedures are related to the issue of recording infectious diseases in the Epidemiological Indications Registry. The answer of respondent A2 can be pointed out here, who, when asked if he/she reported the emergence of an infectious disease in the Epidemiological Indications Registry and if he/she contacted a doctor in relation to it, answered: “*No, I do not record that. No, I do not inform the doctor about that.*”

### 3.3. Information and Knowledge

The analysis of the theme “*Information and knowledge*” revealed that most of the answers could be classified into three categories, namely: information, posters/flyers and training sessions (annually/monthly). Most respondents, i.e., thirteen of them, answered that they were informed about *pediculosis capitis*. One of these respondents stated that he/she received information through public health services, one respondent through professional literature, three respondents by talking to colleagues, four respondents via online sources, six respondents from the health coordinator and most of the respondents, i.e., eight of them, from the kindergarten director (Table 4).

Most of the respondents, i.e., eight of them, pointed out that in the kindergarten where they work there are posters or flyers concerning the signs and symptoms of head lice, and the method of treatment and prevention available to both parents and childcare professionals. However, the answers of respondents A1 and A2 are intriguing. When asked if they make posters or flyers about head lice, they answered: “*We make flyers after the infection has been noticed.*” This points to deficiencies in raising awareness not only among childcare professionals but also among parents before an outbreak of head lice, as well as to the fact that something is done only when a problem arises. Consequently, this raises the question as to whether head lice in modern times are still taboo or whether there is sufficient knowledge concerning this problem, which would make educational activities and training sessions superfluous. It is quite worrying that not enough attention is paid to this infectious disease, which is still a huge problem for both children and parents. A small number of respondents, i.e., three respondents, answered that head-lice-related training sessions were conducted in the kindergarten where they work, but the answer of respondent B is interesting: “*No, we do not have any training sessions specifically related to head lice, but this theme is mentioned in other training sessions. Once a year, a visiting nurse comes to our kindergarten, who conducts training sessions relating to all infectious childhood diseases, and then head lice are also mentioned.*”

### 3.4. Social Issues

After the analysis of the theme “*Social issues*” three categories emerged: information concealment, rejection and lack of resources (Table 5). For the category “information concealment” we obtained data on parents’ attitudes towards pediculosis and the steps kindergarten workers take when faced with this situation. Of the total number of respondents eleven stated that they had encountered information concealment by parents. Eight respondents mentioned that parents withhold such information to avoid embarrassment and social stigma. In this context a comment by respondent B is interesting: “*Yes, I believe parents are hiding it, and what’s more, they are doing some strange things, for example, dying the child’s hair so that lice cannot be seen, and similar stuff.*” Given that most respondents confirmed that parents often conceal information because of embarrassment and social stigma, this helps in answering the question posed in the theme “Knowledge”: it can be concluded that pediculosis is still a taboo subject and a huge problem for many parents. This is confirmed by the answer of respondent I: “*Yes, in a way-we had such a situation where the younger brother was coming to kindergarten, and the older brother was kept at home due to him having head lice. We were not informed about this because the parents were embarrassed.*” Of all the respondents who stated that they had encountered the problem of parents withholding information three thought that the reason was not embarrassment, but rather the fact that they could not get sick leave or days off work because of their child’s pediculosis. For most of them bringing the child to kindergarten was the only option they had.

For the category “rejection” kindergarten workers discussed whether parents refuse to acknowledge the information that their child is infected with head lice and the ways parents deal with this problem. Most respondents, i.e., ten of them, had experienced rejection by parents, which had the following course: when informed about the infection parents come and pick up the child, keep him/her at home for a few days and then, while the child is still infected, bring him/her to kindergarten, claiming that the infection is over. Respondents have often encountered parents’ frustration and even parents who refuse to take care of their child’s infection on their own and want to leave it to kindergarten staff. Most respondents had noticed that parents often do not know enough about pediculosis, leading them to break off the necessary treatment. This is confirmed by the response of K: “*You have parents who claim they have cleaned out the parasites, we admit the child and re-examine them in the presence of parents and health coordinator [and you see this is not the case].*” Among the respondents who had not encountered parents’ rejection there were also positive examples, such as the one given by respondent H: “*The parents volunteer the information and request precautionary measures in order to protect other children. (...) after the scalp has been “cleaned” the child is examined by a doctor, who then provides a note or a written confirmation that the child can go back to kindergarten.*”

The category “lack of resources” is a summary of responses on the conditions in kindergartens in the case of an infection as well as the procedures undertaken when a child is brought in with an ongoing infection. From the total number of respondents two stated that their premises do not allow them to isolate a child; thus, when parents bring a child infected with head lice to kindergarten the staff simply leave this child in their group. Ten respondents said that they call the parents and ask them to come and pick up their child as soon as possible. Only two respondents said that they isolate the child from the group, call the parents and wait for their arrival. On the latter, an answer by respondent M is relevant: “*We remove the child from the group, call the parents and wait for them to pick up the child.*” The remaining respondents had similar answers: they leave the child in the group until the parents arrive, which confirms the fact that a large number of kindergartens do not have adequate premises with which to accommodate children infected with head lice. One respondent said that after calling the parents the staff make a written record, and one stated that they request a doctor’s confirmation that the child is healthy only after he/she has been infected for the second time.

### 3.5. Psychological Issues

After the analysis of the content provided by respondents under the theme “*Psychological issues*” three categories emerged. The main theme was defined on the basis of the following question: “*There is head lice infestation in the kindergarten where you work. Does this fact cause any type of uneasiness in your mind?*” The largest number of respondents (eleven of them) stated that such an occurrence does not make them feel uneasy and that they take it in their stride (Table 6). On the contrary, three respondents stated that they feel mental uneasiness, which manifests itself as a constant feeling of itchiness on the body and scalp. There was an interesting answer by respondent A: *“It was horrible. I even had insomnia because this went on and on. (...) The parents were not cooperative at all, they often quarrelled with us. We (kindergarten staff) bought special shampoo for personal use, washed our hair at home with this special shampoo, we used different sprays-repellents both at work and at home. (...) As soon as I came home, I took off and washed all my clothes. (...) We had a feeling that we were itching all over. We were wearing protective caps to cover our hair. Simply put, there was this constant fear. At that time, my quality of life had diminished.”* Later on, the respondent said that she knew her fear was unfounded and excessive, but she could not shed this feeling.

As stated above, the majority of respondents do not feel any particular uneasiness, i.e., personal psychological effects due to occurrences of head lice infestation in their place of work. However, most of them agree that such an infection is a problem and hard to get rid of, mostly because parents are not cooperative enough. This issue was addressed by the respondent [A]: *“The worst part was cooperation with parents since they didn’t know how to proceed after we called to inform them that their child is infected with head lice. If it happened that we didn’t notice the infection, but they saw it first at home, we were guilty for not noticing it before them.”* The issues of awareness raised among parents and general attitudes of kindergarten staff on the theme of *pediculosis capitis* will be covered further in the Discussion section of the manuscript.

### 3.6. Perception of Pediculosis Capitis

After the analysis of the theme “*Perception of pediculosis capitis*” four categories emerged: taboo subject, poor hygiene, education and low socioeconomic status. These represent the attitudes of respondents towards pediculosis, categorized based on their summarized answers to predetermined questions or elicited opinions that reflect their attitudes. The largest number of respondents (twelve of them) agreed that head lice are still considered taboo. Respondents stated that they have had parents who felt embarrassed when told that their child had head lice or that they needed to talk to them in private. Furthermore, the largest number of respondents (eight) stated that head lice infestation is connected with poor hygiene in children. Respondents stated that the disease is caused by a lack of good hygiene habits, such as changing bedding, washing clothes, cleaning the child’s scalp, hair hygiene in general, etc. Respondent F stated: *“... they do not wash the bedding, towels...*” Most respondents stated that they believe that poor hygiene is a contributing factor in the development of infection, which then spreads to children with good hygiene habits. Respondent L stated: “*Yes, I think that poor hygiene habits are a contributing factor in individuals who catch it first, which then puts children who maintain proper hygiene habits at risk from infection.*” A minority of respondents (four) agreed that the occurrence of *pediculosis capitis* is associated with low socioeconomic status; however, they did not give any examples or offer any explanation for their opinion (Table 7).

The last question, which sought to determine their general attitudes towards pediculosis capitis based on predetermined questions or elicited answers, allowed the respondents to take as much time as they needed and elaborate on their answer. Based on the data obtained the respondents’ attitudes were classified into positive and negative. The largest number of respondents (five) had a positive attitude, i.e., they believed that head lice infestation is a common problem among children and that it should not be a cause for alarm. Furthermore, the respondents stated that there is a need to educate parents and train childcare professionals on how to deal with an outbreak of head lice. Respondent J stated: “*Head lice outbreaks are fairly common in kindergarten (...) It is necessary to educate parents on how to deal with an infection as this requires a coordinated effort of parents, kindergarten staff and health professionals.*” Regarding the need for training childcare professionals, respondent I stated: “*Judging by the reactions of colleagues at the kindergarten where I work who say they are itching all over when an infection occurs and their repulsion over this disease—all this creates panic for no reason. I believe that there is a need to educate staff to correct misconception about this disease.*”

Seven respondents have a positive attitude towards *pediculosis capitis*. They believe that the early diagnosis and treatment of head lice is the best way to manage this disease. In addition, parents should be open to talking about it rather than feel ashamed. Respondent H stated: “*It is important to be open to talking about this Theme and take action as soon as possible to stop the spread of lice infection.*” Of the total number of respondents two had a negative attitude towards pediculosis. They found that an outbreak of head lice causes fear among staff, which negatively affects staff morale as well as their health and well-being. Respondents also stated that in such situations parents expect too much from them, which only contributes to the negative attitude of the staff.

## 4. Discussion

This is the first study that has aimed to concurrently determine the perceived knowledge, attitudes and perspectives of childcare professionals working in kindergartens towards *pediculosis capitis*, i.e., head lice, the procedures and control measures implemented during an outbreak in addition to all the accompanying challenges that they are exposed to. Although studies thus far have not addressed this issue we believe that it is extremely important, as many studies have shown a high prevalence of head lice in kindergarten children. For example, in Bahía Blanca in Argentina the overall prevalence of pediculosis capitis was 42.7%, with girls being affected substantially more frequent than boys (i.e., 53.6% to 28.4%, respectively) and without any difference between socioeconomic classes [11]. Various reports have also addressed the rising rates of head lice in kindergartens in Germany [12], and official data from Croatian National Institute of Public Health also reveal rising rates of ectoparasite infestations [13]. However, qualitative studies similar to ours on this topic have hitherto not been conducted.

By inquiring about the challenges that childcare professionals are faced with in such situations, the behavior of parents and their own knowledge about head lice, as well as training on how to deal with an outbreak of head lice in kindergartens, the data that we have obtained contribute to the understanding of this issue in a wider context. Furthermore, the data on the procedures in the event of a *pediculosis capitis* outbreak reveal that kindergartens lack resources—both staff and adequate premises—to accommodate children infected with lice. Furthermore, additional problems may arise due to the insufficient number of health coordinators; more specifically, a considerable number of respondents report that there is no designated health coordinator in the kindergarten in which they work, which means that kindergarten teachers also serve in the role of health coordinators. Most respondents state that in the case of an infection the entire group is checked for head lice by kindergarten teachers themselves. The issue is compounded with additional challenges that arise due to the lack of adequate training.

Regarding the latter, only three respondents reported that training sessions on how to deal with an outbreak of head lice are provided in the kindergarten where they work. One respondent stated that such training usually concentrates on isolated themes of concern, while two respondents stated that training is provided once a year relating to several areas of concern. This suggests that training that focuses specifically on *pediculosis capitis* is almost never provided, and even when it is it is only mentioned as one of the many infectious diseases discussed. Such a lack of training and (consequently) insufficient knowledge of childcare professionals concerning head lice is reinforced by the fact that health coordinators in some kindergartens provide training only after an outbreak has already occurred. Moreover, this points to the fact that there are not enough health coordinators, and indicates the need to engage epidemiologists and qualified nursing professionals in the training of childcare professionals in kindergartens. One previous study has already demonstrated the potential effectiveness of simple health education initiatives provided by school teaching staff by liaising with parents in order to lower the burden of *pediculosis capitis* in a certain community [14]; we believe there can be a similar role for kindergarten teachers if they have enough knowledge.

Additionally, this brings us to the issue of raising awareness among parents. This is confirmed by the fact that although some of them bring their child to kindergarten confident that they have cleaned their scalp properly, after examination it turns out that the child is still infested. However, most respondents agree that parents often keep it secret that their child is infested because they are embarrassed. This is another indication of the need for raising awareness and increasing educational efforts among parents, since it is evident from our study that they do not have enough information and do not know what to do if their child becomes infested. Their actions actually stem from fear that their child will be shunned or mocked by other children in their group should they be found out. Hence, face-to-face training provided by qualified professionals, who should use visual materials and demonstrations, would help parents understand that head lice infestation is not something to be ashamed of. Our study has also shown that regardless of posters and flyers on bulletin boards in kindergartens parents still do not have enough information about this disease. This raises the question as to whether the problem is caused by the parents’ lack of interest or rather a lack of clarity of the information provided. Whatever the answer may be, it seems that comprehensive awareness campaigns are necessary.

For instance, several respondents state that parents often do not want to take steps to prevent the spread of lice or participate in the treatment of them. A small number of respondents, i.e., three in total, reported having to deal with parents who bought treatment products and brought them to kindergarten, expecting the staff to treat their children for lice. Eleven respondents reported that parents did not share the information that their child was infected. It is interesting that several respondents believed that parents fail to share this information with kindergarten staff not because they feel ashamed, but because they cannot get sick leave or days off from work. This indicates that there is a problem in the health system and highlights the inconsistent practices of employers when it comes to days off in the case of infectious diseases, while some parents do not want to take time off from work for a harmless problem such as lice infection. Furthermore, there is the issue of living arrangements and family support. The data show that most young couples live with their parents [15]; nonetheless, there are those who live in their own homes. Their living arrangements are often correlated with other factors, such as the relationship with their parents (who might be babysitting a sick grandchild) or the fact that parents do not have anyone to leave their child with at home when they are at work. The influence of family structure on certain infectious diseases has already been established in the literature [16].

Most of the respondents reported on a number of challenges in their relationship with the parents when an outbreak occurs. Furthermore, they are faced with challenges on a daily basis due to the lack of training related to *pediculosis capitis*, an insufficient number of staff to adequately care for infected children and an unsatisfactory number of health coordinators to help keep the disease under control. However, despite frequent problems, most of the respondents have maintained a positive attitude. Data were also collected on psychological issues and reactions of the respondents to the occurrence of pediculosis capitis in kindergartens. It is interesting that an outbreak of head lice does not cause uneasiness, worry or fear in the respondents. When they were initially informed about our research theme and overall aims, as well as during the interviews, most of the respondents gave off the impression of negativity—in particular when they were talking about the challenges they are faced with. However, by the end of the interview, when they were asked to describe their general views towards the disease, most answers could be interpreted as a positive attitude. In addition, the respondents were asked to give their opinion about a possible connection between poor hygiene, low socioeconomic status and *pediculosis capitis*—a majority of respondents noted that there was indeed such a connection.

Still, the analysis of the data obtained shows slightly contradicting results related to kindergarten staff attitudes towards head lice. Thus, there are concerns about respondents giving socially desirable answers so as to leave an impression that they feel optimistic about the subject, while being aware of the severity of the problems associated with this infectious disease. Furthermore, given that several respondents mentioned inadequate hygiene among members of the Roma community when asked whether there is a connection between poor hygiene and pediculosis, there is a possibility that some respondents harbored certain prejudices. However, they did not elaborate on this theme even when prompted but concluded that head lice represent a common problem in kindergartens, adding that it should not be a cause for panic and negative feelings. A negative general attitude was more common among the younger respondents with fewer years of service and in the respondents who had children. They were also more commonly concerned about picking up head lice themselves and passing them onto their children. The respondents who had a positive attitude were found to be more commonly concerned about the problems they had to deal with and had a negative general attitude towards the duration of the infection, the duration of treatment and the possibility of reinfection.

To our knowledge there are no publications in the literature that examine the attitudes of kindergarten and preschool teachers towards head lice infestation, but there are several studies that took a deeper dive into school settings. One such research endeavor was a study conducted in a multicultural setting in Perth, Western Australia, with the aim of establishing whether there is a correlation between attitude modification and the management of head lice infestation [17]. The study included primary school children aged 8 to 12. The results show that most of the respondents were not concerned about having head lice because they considered head lice to be a normal part of life and they were confident that they would be able to treat them successfully. Nevertheless, the study found that most children did not tell their parents they were infected, which may be due to indifference or wariness of the possible negative reaction of their parents [17]. Furthermore, the study results show that most parents did not do anything to control the infection, and almost all those who did do something about it did so in relation with their daughters. This shows that stigma is a parental and societal issue rather than a child issue, as only a quarter of the respondents showed negative emotions [17]. Thus, a significant portion of respondents had similar reactions. Although our study included childcare professionals working in kindergartens, we have shown that stigmatization also exists among parents of preschool children and that most of them refuse to take steps to properly address the infection.

Another relevant study, conducted in 2017, included primary school children in Muang Khon-Kaen Province in northeastern Thailand [18]. Its goal was to examine the knowledge, attitudes and preventive practice of parents, teachers and children regarding head lice infestation. Over a period of two months the results in intervention and control groups were monitored using a knowledge, attitude and practice (KAP) questionnaire [18]. The results revealed that the two-month health education program was successful in that it improved knowledge and changed attitudes towards preventive practices—highlighting the importance of education as the majority of respondents reported insufficient awareness about pediculosis to be their main concern [18]. As a result, training sessions for childcare professionals and awareness-raising campaigns for parents would strongly promote the treatment of lice, as well as prevention measures.

Three groups of respondents were surveyed as part of another study conducted in 2019 [19]. The first group included the inhabitants of Moscow and the Moscow region; the second group comprised pharmaceutical specialists who were members of pharmacy organizations in Moscow and the Moscow region; while the third group comprised doctors from dermatovenereology clinics. A qualitative approach and semi-structured questionnaires were used to identify medical and social factors that affect the spread and prevention of *pediculosis capitis*; the collected data were then analyzed using a mathematical approach. The study involved a total of 580 respondents aged 18 to over 61 years that belonged to the first group, of whom 76.5% were women and 23.5% men [16]. The second group consisted of 115 respondents, of whom 51% were qualified pharmaceutical chemists and 49% were pharmacists. The third group consisted of eight specialists in dermatology and venerology. The sociological study found that 62% of the respondents had had *pediculosis capitis*, of whom 44% caught the disease while in primary school, 36.5% in kindergarten, 10% during higher education and 9.5% during holidays. The survey data revealed that a third of the respondents did not tell anyone about the disease, which was one of the main factors that contributed to the development of epidemics [19]. Only one-third of the respondents informed their teachers or the school nurse. In addition, the survey results showed that the reason for nondisclosure was their concern about the reactions of other people. This is explained by the fact that 43% of the respondents experienced a negative attitude when they were infected. Furthermore, 86.5% of the respondents had negative feelings about themselves while they had the disease—of those, 25.5% felt upset about it while 20% felt shy. The survey data show that the majority of the respondents self-treated head lice and did not seek medical attention except when they could not solve the problem on their own [19]. The results of the mentioned study indicate the lack of knowledge among the surveyed population about *pediculosis capitis* and reveal that people still feel ashamed of being infected.

These results are largely consistent with the results of our study. As stated above, the majority of the respondents also reported that parents tend to hide the infestation in their children because of shame, and often fail to inform kindergarten teachers and health coordinators. Hence, to reduce the incidence of head lice it is of the utmost importance to challenge the stereotype of this disease as a disease that affects only the socially disadvantaged and to break down the misconception that it is a sign of poor personal hygiene. The fact is that children from any social background can be infected, but most people who have had it experienced negative feelings as well as attitudes and did not want other people to know they were (or had been) affected. The present study also reveals that, in spite of the fact that quite some time has passed since they had the disease during their own childhood, the negative attitude towards it may stay with them throughout their adult life. Interestingly, the negative reactions of parents and professionals to head lice may be reflected in the drawings of kindergarten children [20]; hence, the emotional toll of this problem also has to be taken into account.

## 5. Conclusions

Our study identified five thematic areas after we conducted thorough thematic analysis: (1) prevention and control measures for managing head lice, (2) information and knowledge, (3) social issues, (4) psychological issues and (5) disease perception. Each of these themes also had specific emerging categories based on participants’ responses. In the prevention and control category several epidemiological perspectives emerged within four categories: monitoring the disease within the kindergarten community, notice to parents of all children in kindergarten, scalp examination and isolation. The information and knowledge theme included information *sensu stricto*, but also posters/flyers in line with health promotion as well as annual/monthly training sessions. The theme of social issues emphasized information concealment, rejection and a lack of resources, while the theme of psychological issues included mental uneasiness, worry and fear as the dominant categories. Finally, the perception of *pediculosis capitis* as a theme identified the notion of the disease as a taboo subject, linked with poor hygiene, lower socioeconomic status and the lack of adequate education. In conclusion, our findings have implications for quotidian practice and highlight the need for the introduction of tailored public health measures in order to address the most vulnerable populations, particularly kindergarten children. Further research endeavors in this field will have to address control measures implemented by parents as well as specific protocols for the return of children to classes.

## Figures and Tables

**Table 1:** Demographic profile of the childcare professionals included in the study.

Respondent ID	Sex	Age	Educational Attainment	Overall Length of Service	Group of Children	County	Location
A	F	25	Bachelor’s degree	1 year and 4 months	Nursery (1–3 years)	Međimurska County	Čakovec
B	F	38	Bachelor’s degree	15 years	Preschoolers (6–7 years)	Varaždinska County	Petrijanec
C	F	24	Bachelor’s degree	1 year and 1 month	Mixed-age (2–4 years)	Međimurska County	Čakovec
D	F	29	Bachelor’s degree	4 years and 2 months	Nursery (toddlers)	Varaždinska County	Sračinec
E	F	42	Bachelor’s degree	20 years	4- to 5-year-olds	Varaždinska County	Sračinec
F	F	45	Bachelor’s degree	19 years	Preschoolers (6–7 years)	Varaždinska County	Sračinec
G	F	41	Bachelor’s degree	2 years and 9 months	Preschoolers (6–7 years)	Varaždinska County	Sračinec
H	F	46	Bachelor’s degree	17 years	Mixed-age (3–7 years)	Međimurska County	Čakovec
I	F	22	Bachelor’s degree	6 months	Mixed-age (4–7 years)	Varaždinska County	Cestica
J	F	31	Bachelor’s degree	8 years	4- to 5-year-olds	Varaždinska County	Črešnjevo
K	M	43	Master’s degree in Education	14 years	5- to 7-year-olds	Varaždinska County	Varaždin
L	F	56	Bachelor’s degree	23 years	4- to 5-year-olds	Varaždinska County	Varaždinske Toplice
M	F	41	Bachelor’s degree	14 years	Preschoolers (6–7 years)	Varaždinska County	Varaždinske Toplice
N	F	56	Bachelor’s degree	25.5 years	Preschoolers (6–7 years)	Varaždinska County	Varaždinske Toplice

Abbreviations: F—female; M—male.

**Table 2:** Demographic profile of the employed, on-site health coordinators included in the study.

Respondent ID	Sex	Age	Educational Attainment	Overall Length of Service	Number of Working Hours Daily	County	Location
A1	F	25	Bachelor’s degree	1 year	8 h (or less)	Međimurska County	Čakovec
A2	F	26	Bachelor’s degree	3 years	8 h	Varaždinska County	Sračinec
A3	F	35	Bachelor’s degree	5 years	8 h	Varaždinska County	Črešnjevo

Abbreviations: F—female.

**Table 3:** Thematic analysis of categories and verbatim quotes for theme one: “*Prevention and control measures for managing head lice*”.

Main Theme	Categories	Respondent ID	Example
Prevention and control measures for managing head lice	Monitoring the disease within the kindergarten community	A, B, C, D, E, F, G, H, I, J, K, L, M, N, A1, A2	*“Each child is examined separately.”* (J)
	Notice to parents of all children in kindergarten	A, B, C, D, E, F, G, I, J, K, L, M, N, A1, A2	*“We inform all parents. A notice is placed on the bulletin board and in the Facebook group through which we communicate with all parents.”* (B) *“Yes, parents are informed both in writing and orally.”* (J)
	Scalp examination	H, I, J, A1	*“The health coordinator examines each child’s scalp.”* (H) *“The child is examined; if live lice or nits are found, then the parents are called, but if there are only dead lice that have not been removed, then the health coordinator or childcare professionals clean the child’s hair and the child stays in kindergarten.”* (I)
	Isolation	A, M, A1	“*The child is isolated from the group, they are mostly taken to the bathroom, they must put a cap on, the parents are called, and together with the childcare professional they wait for their arrival.*” (A)

**Table 4:** Thematic analysis of categories and verbatim quotes for theme two: “*Information and knowledge*”.

Main Theme	Categories	Respondent ID	Example
Information and knowledge	Information	A, C, D, E, F, G, H, I, J, K, L, M, N, A1, A2	*“… we provide information if requested by childcare professionals.”* (A1) *“Online sources, the kindergarten director, other childcare professionals.”* (D)
	Posters/flyers	A, E, F, G, H, I, K, K, A1, A2	*“I make posters that are placed on the bulletin board for parents, and hence for childcare professionals, too.”* (A2) *“There are posters on the bulletin board and flyers.”* (I)
	Training sessions (annually/monthly)	I, J, K, A2	*“Yes, I conduct training sessions after infestation.”* (A2) *“Yes, as necessary.”* (K)

**Table 5:** Thematic analysis of categories and verbatim quotes for theme three: “*Social issues*”.

Main Theme	Categories	Respondent ID	Example
Social issues	Information concealment	A, B, D, E, F, I, J, K, L, M, N	*“Yes, we have had a few cases where parents withheld information because they were embarrassed.”* (A) *“Parents sometimes hide the fact that they have noticed head lice because they cannot miss work for this.”* (E)
	Rejection	A, B, D, E, F, G, I, K, M, N	*“... I feel that in these situations parents expect too much from us. We even had parents bringing electric combs and asking us to clean their children’s hair because they have no time to deal with this at home.”* (B) *“Parents are confident that they have cleaned their child’s scalp properly and bring the child to kindergarten.”* (I)
	Lack of resources	B, I	*“... we had a case where the parents brought the child to kindergarten claiming that the child is now free from lice (…) They were very upset with us and we had to accept the child and keep it in the group since we don’t have extra rooms or enough employees to isolate a child and have one person spend the day with this one child.”* (B)

**Table 6:** Thematic analysis of categories and verbatim quotes for theme four: “*Psychological issues*”.

Main Theme	Categories	Respondent ID	Example
Psychological issues	Mental uneasiness	A, K	*“It was horrible. I even had insomnia because this went on and on. (...) a feeling that we were itching all over. We were wearing protective caps to cover our hair.”* (A)
	Worry	B	*“... of course I am worried I might become infected – I have a child of my own and I am afraid I might pass it on to my child.”* (B)
	Fear	A	*“... Simply put, there was this daily fear.”* (A)

**Table 7:** Thematic analysis of categories and verbatim quotes for theme five: “*Perception of pediculosis capitis*”.

Main Theme	Categories	Respondent ID	Example
Perception of *pediculosis capitis*	Taboo subject	A, B, C, D, G, H, I, J, K, L, M, N	*“Yes, because parents feel uncomfortable when we tell them that their child has head lice.”* (H)
	Poor hygiene	B, D, E, F, G, I, M, N	*“I find that the conditions in which some children live are a factor that contributes to the development of infection. Parents do not check their children for head lice, they don’t change the bedding, they don’t wash the clothes and it’s only normal that things like that happen.”* (B) *“Most often, it is Roma children who become infected first. We know that some of them live in poor hygienic conditions at home. The infection then spreads to other children...”* (I)
	Education	F, G, I, J, L	*“Head lice should be treated as any other disease. I think parents should be educated on how to deal with it and how to help their child in this situation.”* (L)
	Low socioeconomic status	B, E, I, K	*“I find that head lice infestation is often associated with low socioeconomic status.”* (K)

## Data Availability

The data presented in this study are available on request from the corresponding author. The data are not publicly available due to privacy issues.
